# Supplementary material for: Reduction in and Preventive Effects for Oral-Cancer Risk with Antidepressant Treatment
Source: J Pers Med. 2021 Jun 23;11(7):591. doi: 10.3390/jpm11070591 (PMC8307663; doi:10.3390/jpm11070591)
Supplement: Supplementary file 1 [file jpm-11-00591-s001.zip › jpm-1228362-supplementary.pdf]

Supplementary figures and tables

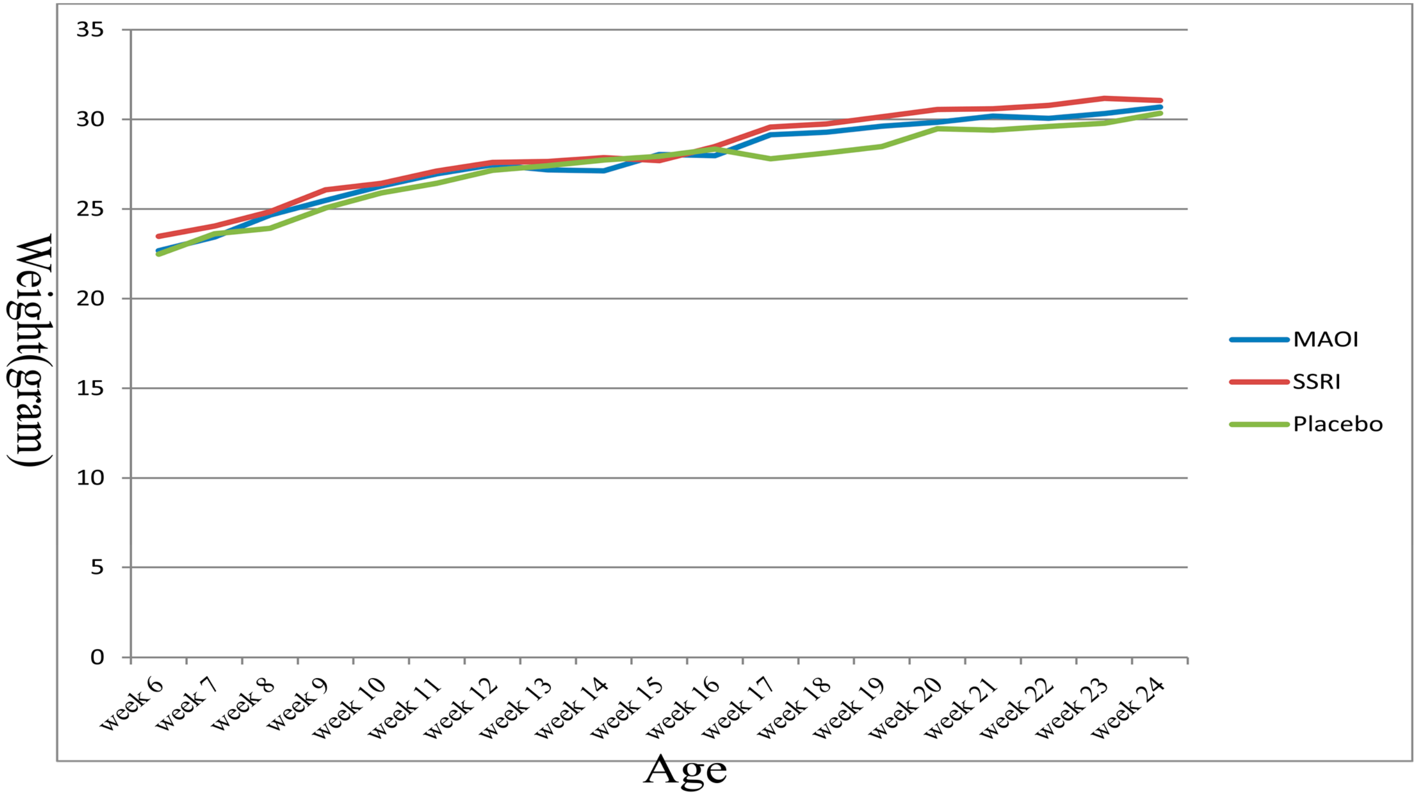

Supplementary Figure S1. Comparison of body weight changes with long-term areca nut treatment among MAOI, SSRI and Placebo groups.

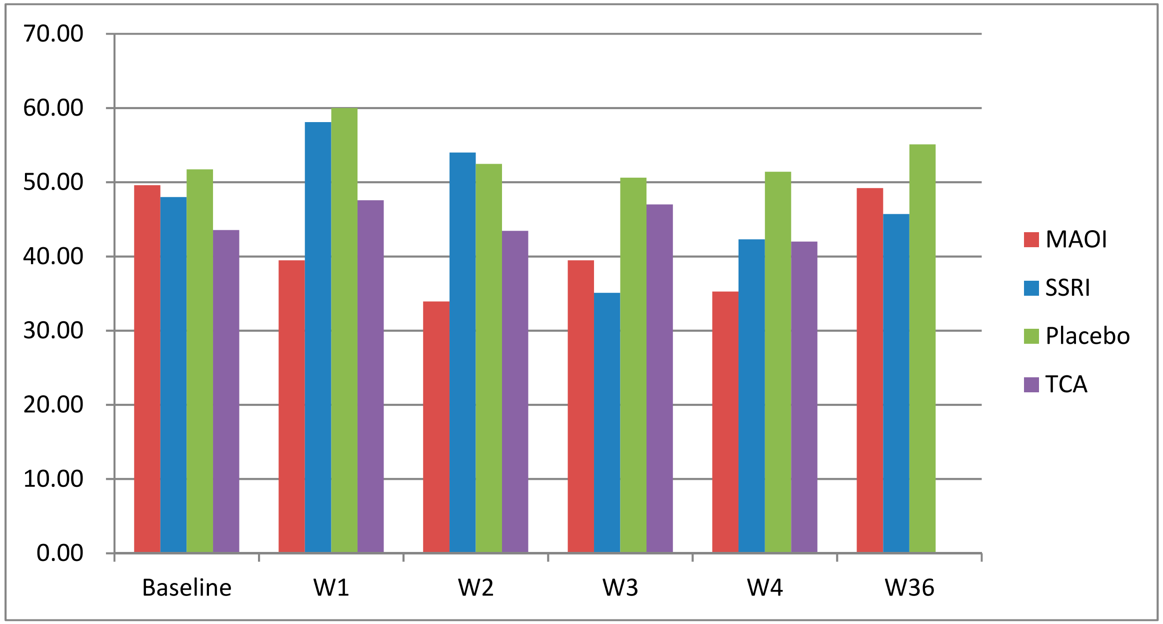

Supplementary Figure S2 Comparison in consumption amount of areca nut water after antidepressants treatment in mouse mode (follow-up 4 weeks and 36 weeks). TCA group was not significantly decreased in consumption amount of areca nut compared to placebo group. The follow-up period was halted at fourth weeks after treatment.

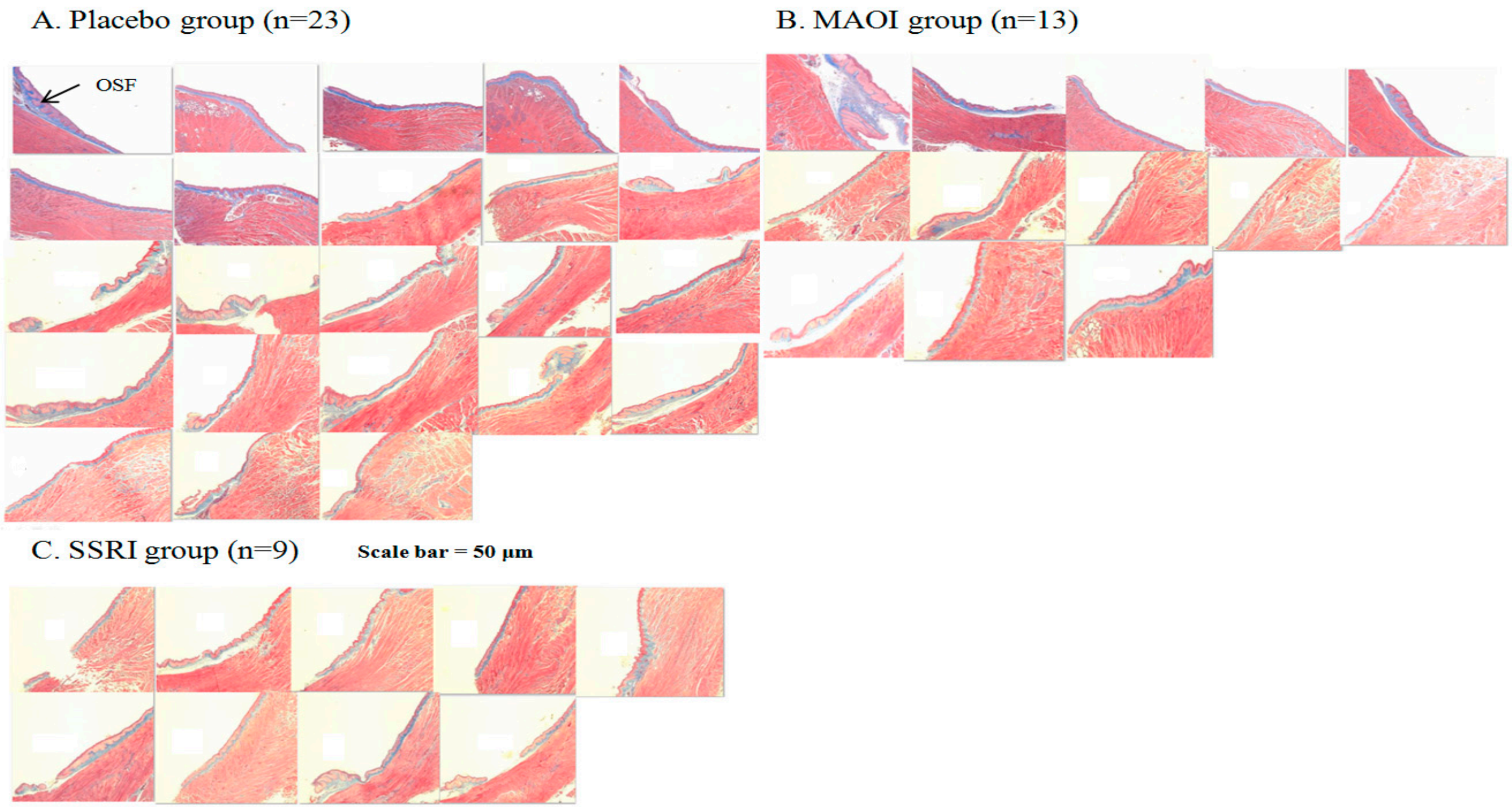

Supplementary Figure S3. H&E trichrome of oral submucous fibrosis in the mouse tongue tissue. Photomicrograph showing oral submucous fibrosis (Blue color) in mouse tongue tissue. Scale bar = 50 µm. A: placebo group (n=23), B. MAOI group (n=13), C. SSRI group (n=9).

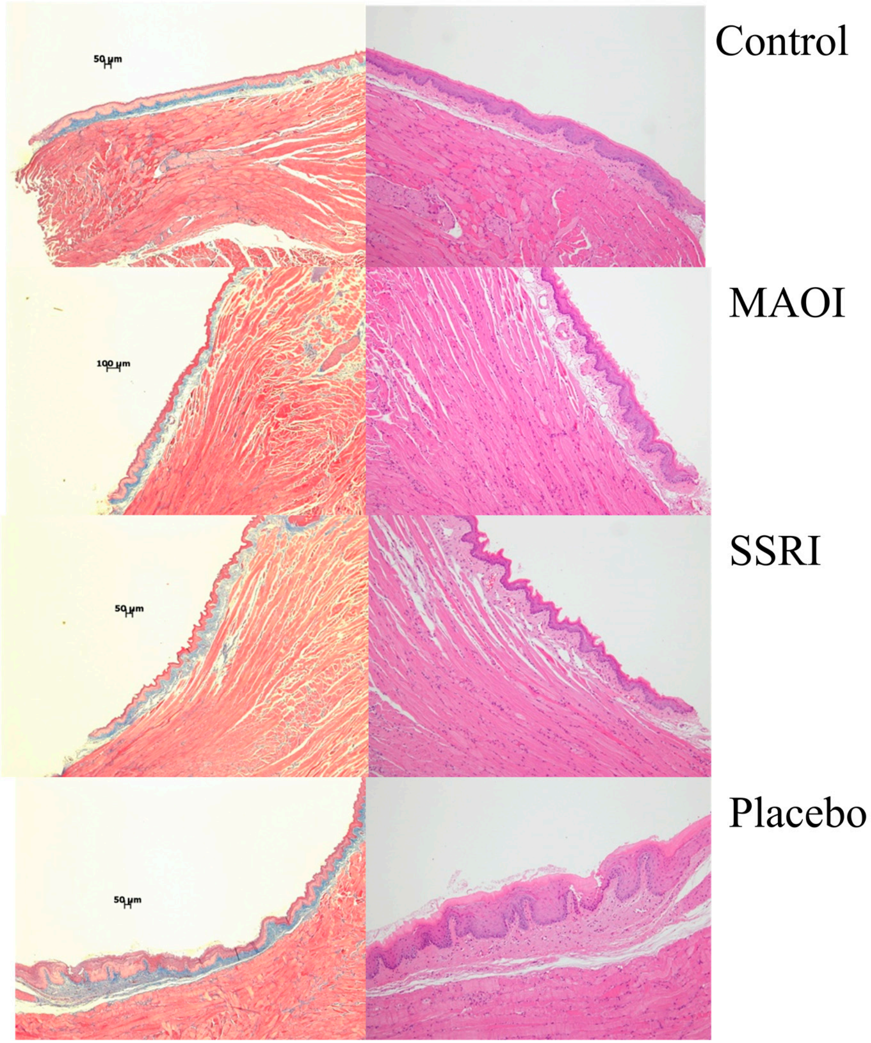

Supplementary Figure S4 H&E trichrome of oral submucous fibrosis in the selective and paired mouse tongue tissue

Supplementary Table S1. Changes in consumption amount (averaged ml/ weekly) of area nut water after antidepressants treatment analyzed by GEE model

				MAOI				SSRI			
**Scale**	Placebo, Mean ± SD (*n*)	MOAI ± SD (n)	SSRI, Mean ± SD (*n*)	Estimate	SEM	Z	p	Estimate	SEM	Z	p
Baseline	21.7±4.92(15)	19.6±4.19(15)	18±11.6(10)								
Week 1-2	26.2±4.19(15)	6.7±8.96(15)	26.05±11.4(10)	0.15	0.07	2.29	0.02	0.08	0.04	1.85	0.06
Week 3-4	21.0±6.30(15)	7.4±5.08(15)	8.2±4.3(10)	-0.02	0.03	-0.79	0.43	-0.01	0.03	-0.50	0.62
Week 36	25.1±5.84(15)	19.2±4.15(15)	15.7±5.46(10)	0.03	0.01	1.94	0.05	0.03	0.01	2.01	0.04
Drug											
Week1-2 × drug				-0.38	0.09	-4.09	<.0001	0.07	0.08	0.90	0.37
Week3-4× drug				-0.27	0.08	-3.35	<.0001	-0.21	0.11	-2.02	0.04
Week 36× drug				-0.03	0.02	-2.32	0.02	-0.08	0.08	-0.99	0.32
